# Comparative analysis of diosgenin in *Dioscorea* species and related medicinal plants by UPLC-DAD-MS

**DOI:** 10.1186/1471-2091-15-19

**Published:** 2014-08-09

**Authors:** Tao Yi, Lan-Lan Fan, Hong-Li Chen, Guo-Yuan Zhu, Hau-Man Suen, Yi-Na Tang, Lin Zhu, Chu Chu, Zhong-Zhen Zhao, Hu-Biao Chen

**Affiliations:** 1 School of Chinese Medicine, Hong Kong Baptist University, Hong Kong Special Administrative Region, PR China; 2 Guangxi Botanical Garden of Medicinal Plant, Nanning, Guangxi 530023, China; 3 Department of Chemistry, Lanzhou University, Lanzhou 730000, China; 4 The State Key Laboratory of Quality Research in Chinese Medicine, Macau University of Science and Technology, Macau, China; 5 College of Pharmaceutical Science, Zhejiang University of Technology, Hangzhou 310014, China

**Keywords:** Diosgenin, UPLC-DAD-MS, *Dioscorea*, Medicinal plants, Quality evaluation

## Abstract

**Background:**

*Dioscorea* is a genus of flowering plants, and some *Dioscorea* species are known and used as a source for the steroidal sapogenin diosgenin. To screen potential resource from *Dioscorea* species and related medicinal plants for diosgenin extraction, a rapid method to compare the contents of diosgenin in various plants is crucial.

**Results:**

An ultra-performance liquid chromatography (UPLC) coupled with diode array detection (DAD) and electrospray ionization mass spectrometry (ESI-MS) method was developed for identification and determination of diosgenin in various plants. A comprehensive validation of the developed method was conducted. Twenty-four batches of plant samples from four *Dioscorea* species, one *Smilax* species and two *Heterosmilax* species were analyzed by using the developed method.

The present method presented good sensitivity, precision and accuracy. Diosgenin was found in three *Dioscorea* species and one *Heterosmilax* species, namely *D. zingiberensis*, *D. septemloba*, *D. collettii* and *H. yunnanensis*.

**Conclusion:**

The method is suitable for the screening of diosgenin resources from plants. *D. zingiberensis* is an important resource for diosgenin harvesting.

## Background

*Dioscorea* (yam) is a genus of over 600 species of flowering plants in the family Dioscoreaceae, native throughout the tropical and warm temperate regions of the world. Apart from the traditional importance as starchy staple food (such as *D. opposita, D. alata* and *D. japonica*) [1], some *Dioscorea* species are known and used as a source for the steroidal sapogenin diosgenin, a precursor for the synthesis of steroid drugs [2]. However, the reserves of wild *Dioscorea* plants continue to decline, because of the extensive harvesting and increasingly ecological damage [3]. Yet the failure of achieving fully chemical synthesis of steroids until now again made *Dioscorea* a very attractive source for steroidal precursors.

China and Mexico are the top two countries with the richest yam resource in the world, the yield of diosgenin accounts for 67% of world production [4]. As far as we know, there are 49 species of the genus *Dioscorea* distributed in China, and some related medicinal plants are confusedly used in the folk medicine [5-7]. In recent decades, diverse medicinal plants from the genera *Dioscorea* and *Smilax*, and even minor species from the genus *Heterosmilax*, have been documented under the name “Bixie” or a very similar name as folk medicines in different areas of China [8-10]. However, it has not been reported whether they can be used as a source for steroidal sapogenin diosgenin. This situation limits the comprehensive utilization of these plant resources. Therefore, to overcome the shortage of raw materials and to support sustainable development of the pharmaceutical industry associated with diosgenin, screening of potential plants resource from *Dioscorea* species and related medicinal plants for diosgenin extraction is urgently needed. Undoubtedly, to develop a rapid method which can compare the contents of diosgenin in various plants remains the primary task.

To solve this problem, some studies have attempted to analyze diosgenin in these medicinal plants by using colorimetry [11,12], thin layer chromatography (TLC) [13,14] and high performance liquid chromatography (HPLC) [15-17]. However, the specificity and precision of colorimetry and TLC were unsatisfactory. The current HPLC method also seems time-consuming. Moreover, the reported contents only partially contributed to diosgenin screening from the genus, because the analytical methods varied considerably. Ultra performance liquid chromatography (UPLC) is a relatively new technique giving new possibilities in saving of analysis time and solvent consumption. UPLC combined with photodiode array detection (PAD) and mass spectrometric (MS) techniques can provide online ultraviolet (UV) and MS information for each analyte in a chromatogram, and this has been proven to be a powerful tool for the rapid qualitative and quantitative analyses of the constituents in botanic extracts and herbal products [18-20].

In the present study, a UPLC-DAD-MS method was developed for the analysis of diosgenin in four *Dioscorea* species and related medicinal plants including one *Smilax* species and two *Heterosmilax* species. The results demonstrated that our method is highly precise and accurate, which is suitable for the screening of diosgenin resources from plants. Among the tested plant samples, diosgenin was found in three *Dioscorea* species and one *Heterosmilax* species, namely *D. zingiberensis*, *D. septemloba*, *D. collettii* and *H. yunnanensis*. Our reserch demonstrated that *D. zingiberensis* is an important resource for diosgenin harvesting.

## Methods

### Materials

The sources of the tested samples are listed in Table 1. Identity of the samples was confirmed by Dr. Hubiao Chen, School of Chinese Medicine, Hong Kong Baptist University [21,22]. Corresponding voucher specimens were deposited in the Chinese medicines center, Hong Kong Baptist University. (No. DYSY for *D. zingiberensis*, No. MBX for *D. septemloba*, No. CRSY for *D. collettii*, No. SY for *D. opposita*, No. GYBQ for *Smilax glbra*, No. XBQ for *Heterosmilax japonica*, No. DZXBQ for *H. yunnanensis*, respectively).

**Table 1:** **Contents of diosgenin in *Dioscorea *** **species and related medicinal plants**

**Species**	**Source and year of harvest**	**Diosgenin (mg/g)**
*D. zingiberensis*	1) Lingbao, Henan, China (2011)	11.47 ± 0.43
	2) Huaihua, Hunan, China (2010)	11.23 ± 0.29
	3) Shanxi, China (2010)	18.35 ± 0.52
	4) Yuanling, Hunan, China (2012)	19.52 ± 0.51
	5) Hubei (2012)	13.20 ± 0.36
	6) Anhua, Hunan, China (2012)	11.30 ± 0.36
	7) Chenxi, Hunan, China (2012)	16.58 ± 0.57
	8) Xupu, Hunan, China (2012)	14.36 ± 0.34
	9) Zhongfang, Hunan, China (2012)	19.00 ± 0.29
	10) Shanxi,China (2012)	8.67 ± 0.28
*D. septemloba*	1) Hunan, China (2011)	0.78 ± 0.02
	2) Hunan, China (2011)	1.12 ± 0.02
	3) Hunan, China (2011)	1.11 ± 0.04
	4) Hunan, China (2011)	1.18 ± 0.02
	5) Chengdu, China (2012)	1.14 ± 0.03
*D. collettii*	1) Guizhou, China (2012)	13.19 ± 0.26
*D. opposita*	1) Henan, China (2011)	ND
	2) Henan, China (2011)	ND
	3) Jiaozuo, Henan, China (2011)	ND
*Smilax glabra*	1) Shek Lei Pui Reservoir, Hong Kong (2012)	ND
	2) Duyun, Guizhou (2011)	ND
	3) Kam Shan Country Park, Hong Kong (2012)	ND
*Heterosmilax japonica*	1) Ceheng, Guizhou, China (2010)	ND
*H. yunnanensis*	1) Ceheng, Guizhou, China (2010)	0.05 ± 0.002

All values are expressed as mean ± standard deviation (n = 3).

ND = Not detected.

### Reagents and chemicals

**Figure 1:**
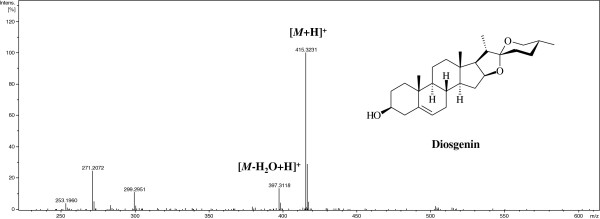
Chemical structure and MS spectrum of diosgenin in positive ion mode.

Acetonitrile of HPLC grade and methanol of analytical grade were purchased from Lab-scan (Bangkok, Thailand). Hydrochloric acid (c.a. 37%) and chloroform purchased from Lab-scan (Bangkok, Thailand) were used for the acid hydrolysis and partition of samples. Water was purified using a Milli-Q water system (Millipore; Bedford, MA, USA).The standard compound of diosgenin was purchased from Phytomarker Ltd. (Tianjin, China). The purity of diosgenin was determined to be more than 98% by normalization of the peak area detected by UPLC-DAD. The chemical structure of diosgenin is shown in Figure 1.

### UPLC–DAD–MS instrumentation and conditions

A Waters Acquity™ ultra performance liquid chromatography (UPLC) system (Waters Corp., Milford, USA) with photodiode array detection (PAD), was hyphenated to a Bruker MicrOTOFQ system by an electrospray ionization (ESI) interface (Bruker Daltonics, Bremen, Germany) for chromatographic and mass spectrometric (MS) analysis. Data analysis was conducted using DataAnalysis software version 4.0 (Bruker Daltonics). For chromatographic separation, a Waters BEH C_18_ column (1.7 μm, 2.1 × 100 mm) with a VanGuard™ pre-column (BEH, C_18_, 1.7 μm, 2.1 × 5 mm) was used. The mobile phase consisted of 0.1% formic acid in water (A) and 0.1% formic acid in acetonitrile (B) using an isocratic elution of 82% (B) in 0–10 min. The solvent flow rate was 0.3 mL/min, the column temperature was set to 40°C, and the detection wavelength was 203 nm. The conditions of MS analysis in the positive ion mode were as follows: drying gas (nitrogen), flow rate, 8 L/min; gas temperature, 180°C; scan range, 50–1600 m/z; end plate offset voltage, -500 V; capillary voltage, 4500 V; nebulizer pressure, 2.5 Bar.

### Preparation of standard and sample solutions

The stock solution of diosgenin (1000 mg/L) were prepared in methanol and stored in the refrigerator. The working solutions were prepared by appropriate dilution of the stock solutions with methanol, and the resulting concentrations were 1, 5, 10, 50, 100, 200, and 500 mg/L, respectively. An aliquot of 5 μL for each calibration standard solution was injected for UPLC analysis.

The preparation of sample solution was prescribed in our study [23]. Briefly, plant materials were cut into small pieces and mixed thoroughly. A representative portion of the sample pieces was ground into a powder that passed through a 20 mesh (0.9 mm) sieve. Herbal sample powder (0.5 g) was extracted with 15 mL of methanol by means of sonication at room temperature for 0.5 h. The operations were repeated two times, and the residue was washed with 5 mL of fresh extraction solvent. Total extracts were combined in a 50-mL volumetric flask, which was filled up to the calibration mark with extraction solvent. The sample solution of 25 mL for glycoside assay was transferred into a round-bottomed flask and evaporated to dryness by rotary evaporation under vacuum at 60°C. An amount of 20 mL hydrochloric acid solution (10%) was added to the residue, heated to hydrolyse on a water bath for 30 min, cooled, and washed with 10 mL of chloroform each time for three times. After the combined mixture was extracted and partitioned, the lower layer (chloroform layer) was collected and the upper layer was extracted with an additional 30 mL of chloroform once. The combined chloroform layer was evaporated to dryness and the residue was dissolved by an appropriate amount of methanol, transferred to a 25 mL volumetric flask and the volume was made up to the calibration mark with methanol. The extracts were then filtered through a syringe filter (0.2 μm). An aliquot of 5 μL solution was injected for UPLC–MS analysis.

### Assay validation and sample determination

Calibration curve is established for each standard compound. *D. zingiberensis* of batch 1 from Lingbao was chosen for method validation. Repeatability was evaluated in intra- and inter-day assays. Recovery of all the quantified constituents was determined by sample in different concentration levels using a mixture of standards with 50, 100 and 200% of the quantified levels of constituents in the samples. All plant samples collected from various regions were analyzed using the present method.

## Results and discussion

### Optimization of extraction and hydrolysis conditions

Various extraction methods (e.g. reflux, sonication), solvents (e.g. different concentrations of methanol or ethanol) and times of extraction were evaluated to obtain maximum extraction efficiency. The results demonstrated that there was no significant difference in the yield of analytes between sonication and reflux, and exhaustive extraction was achieved by sonication with methanol three times for 30 min each time. Hydrolysis conditions were optimized according to the method mentioned in the Chinese Pharmacopeia (2005 edition). Hydrochloric acid rather than sulfuric acid was used because of its low boiling point and good volatility. Chloroform was used as the extraction solvent after hydrolysis because of incompatibility with water and good solubility with diosgenin. The other parameters, including solvent volume, acid concentration, hydrolysis time and extraction times, were further tested. The final conditions are presented in detail in the section of “*Preparation of standard and sample solutions*”.

### Chromatographic conditions and online ESI/MS identification

**Figure 2:**
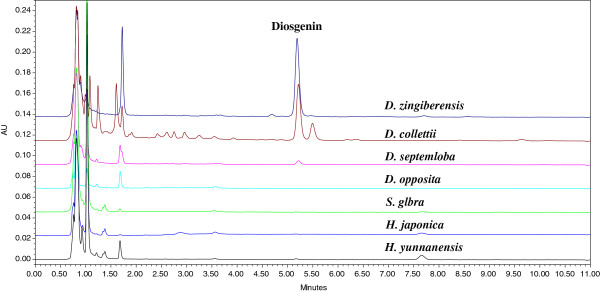
Typical UPLC chromatograms of plant samples using DAD at 203 nm.

The chromatographic conditions such as column, mobile phase and gradient elution were optimized in the preliminary test to achieve better symmetry and shorter retention time of chromatographic peak. After comparing Waters HSS C_18_ column and BEH C_18_ column, BEH C_18_ column was selected as analytical column, because a symmetrical peak of diosgenin was obtained. Mobile phase, such as acetonitrile-water and methanol-water, was compared on a HSS C_18_ column and BEH C_18_ column at different temperatures. The results showed that satisfactory separation could best be obtained by eluting plant samples on a BEH C_18_ column at 40°C using an isocratic flow of acetonitrile and water within 10 min. After comparing the UV chromatograms recorded at wavelengths from 190 to 500 nm, 203 nm was chosen as the wavelength to determine diosgenin in various plants (Figure 2).

The mass spectrometric conditions were optimized in both positive and negative ion modes; the positive ion mode was found to be more sensitive. In order to promote the formation of quasi-molecular ions [*M* + H]^+^ in MS analysis, 0.1% formic acid was used in the mobile phase. MS offset voltage was further adjusted to generate characteristic fragments of diosgenin. A typical MS chromatogram and MS characteristics obtained from the analysis of a plant sample is shown in Figure 1 and Table 2.

**Table 2:** Mass spectral characteristics of diosgenin

**Retention time (min)**	**Analyte**	**Formula**	**Selected ion**	***m/z*** **calculated**	***m/z*** **observed**	**Error (ppm)**
5.2	Diosgenin	C_27_H_43_O_3_	[M + H]^+^	415.3212	415.3231	1.9
		C_27_H_41_O_2_	[M-H_2_O + H]^+^	397.3107	397.3118	1.1

### Validation of the analysis method

The linearity, regression and linear ranges of diosgenin were summarized in Tables 3, 4 and 5. The data indicated good linearity between concentrations and peak areas of the analytes within the test ranges (*R*^*2*^ ≥ 0.999). Based on visual evaluation with a signal-to-noise ratio of about 3:1 and 10:1, the LOD and LOQ of diosgenin were found to be 0.3 and 0.8 ng, respectively. Therefore, the system was considered to be sensitive for the analysis. The RSD of intra and inter day diosgenin variations did not exceed 3.73% and 2.16%, respectively. The established method also had acceptable accuracy with average recovery of 99.43% for diosgenin. As to stability test, the RSDs of the peak areas for diosgenin detected within 24 h were lower than 4.57%. All these results demonstrated that the developed UPLC method was sufficiently reliable and accurate and is therefore suitable for quantification of diosgenin in herbal extract.

**Table 3:** Linearity factors, LOD and LOQ of diosgenin

**Analyte**	**Equation**	**Range (mg/L)**	***R***^***2***^	**LOD (ng)**	**LOQ (ng)**
Diosgenin	y = 2259.94× + 158.19	1–500	0.9998	0.3	0.8

**Table 4:** Precision, repeatability and stability of the method

**Analyte**	**Precision RSD (%,** ***n*** **= 6)**	**Repeatability**							**Stability RSD (%,** ***n*** **= 6)**
		**Day 1**		**Day 2**		**Day 3**		**Inter-day RSD (%)**	
		**Determined (mg/g)**	**RSD (%)**	**Determined (mg/g)**	**RSD (%)**	**Determined (mg/g)**	**RSD (%)**		
Diosgenin	2.40	11.01 ± 0.24	2.18	11.47 ± 0.43	3.73	11.10 ± 0.22	2.01	2.16	4.57

**Table 5:** Recovery of diosgenin in method accuracy test

**Analyte**	**50%**		**100%**		**200%**		**Average**	
	**Recovery (%)**	**RSD (%)**	**Recovery (%)**	**RSD (%)**	**Recovery (%)**	**RSD (%)**	**Recovery (%)**	**RSD (%)**
Diosgenin	102.47 ± 2.54	2.48	100.51 ± 2.53	2.51	95.30 ± 3.22	3.38	99.43	3.73

Values shown are mean ± standard deviation (*n* = 3).

### Sample analysis

The present method was successfully applied to the quantification of diosgenin in herbal samples from different localities. The results are summarized in Table 1. The results demonstrated a variation in the contents of the quantified constituents in testes samples. Such variations may presumably be attributed to differences in plant resources.

The data demonstrated that *D. opposita*, *Smilax glbra*, *Heterosmilax japonica* do not contain diosgenin. In China, *D. opposite* is mainly consumed as starchy staple food [24,25], while *S. glbra* and *H. japonica* are mainly used as folk medicine [26-28]. *D. collettii*, *D. septemloba* and *H. yunnanensis* contain diosgenin, but the number of the tested samples is still relatively small. Further investigation based on a large number of samples is needed. It’s worth noting that the content of diosgenin is up to 19.52 mg/g in *D. zingiberensis*, and the average content of diosgenin in ten batches is 14.37 mg/g. The abundance of diosgenin in *D. zingiberensis* is higher than those in *D. nipponica* (12.52 mg/g) and *D. panthaica* (5.29 mg/g) [23], the other two *Dioscorea* species used for diosgenin extraction in China. Moreover, *D. zingiberensis* is widely distributed in China, including Hennan, Hubei, Hunan, Shanxi, Gansu and Sichaun provinces. Therefore, *D. zingiberensis* is an important plant resource for diosgenin acquisition.

## Conclusions

A UPLC-DAD-MS method was developed for comparative analysis of diosgenin in *Dioscorea* species and related medicinal plants. With respect to already existing reports, the present method, hyphenating UPLC to both DAD and MS techniques, has the advantages of faster, more accurate, and comparative analysis of various herbal samples. By comparing the diosgenin contents, the results demonstrated that *D. zingiberensis* is one of important plant resources for diosgenin harvesting. Our study significantly contributes to the research and development of medicinal plants, especially for the screening of resource plants for diosgenin.

## Abbreviations

UPLC: Ultra performance liquid chromatography; DAD: Diode array detection; ESI: Electrospray ionization; MS: Mass spectrum; TLC: Thin layer chromatography; HPLC: High performance liquid chromatography.

## Competing interests

The authors declare that they have no competing interests.

## Authors’ contributions

TY and LLF initiated and all authors designed the study. The sample extraction was conducted by YNT and HMS. The optimization of experimental conditions was performed by LZ and CC. The data analysis was conduct by HLC and GYZ. ZZZ and HBC drafted the manuscript. All authors contributed to data analysis and manuscript finalization. All authors read and approved the final manuscript.
